# One-year Allograft and Patient Survival in Renal Transplant Recipients Receiving Antiplatelet Therapy at the Time of Transplantation

**Published:** 2018-02-01

**Authors:** T. Benkö, M. Gottmann, S. Radunz, A. Bienholz, F. H. Saner, J. W. Treckmann, A. Paul, D. P. Hoyer

**Affiliations:** 1 *Department of General, Visceral and Transplantation Surgery, University Hospital Essen, University Essen-Duisburg, Germany*; 2 *Department of Nephrology, University Hospital Essen, University Essen-Duisburg, Germany*

**Keywords:** Platelet aggregation inhibitors, Kidney transplantation, Graft survival, Postoperative period, Postoperative complications

## Abstract

**Background:**

Antiplatelet therapy is common in patients on the waiting list for kidney transplantation.

**Objective:**

To evaluate the incidence of post-operative bleeding in patients with antiplatelet therapy undergoing kidney transplantation and analyze the impact on the outcome.

**Methods:**

We studied all patients with concomitant antiplatelet therapy undergoing kidney transplantation in our center from January 2007 to June 2012. Data were collected by chart review. Univariate and multivariate logistic regression and Cox proportional hazard model were used to identify risk factors for the long-term outcome.

**Results:**

Of 744 kidney transplant recipients during the study period, 161 received oral antiplatelet therapy and were included in the study. One-third of the patients demonstrated signs of bleeding, half of which requiring surgical treatment. Coronary artery disease, deceased donor kidney transplantation, and dual antiplatelet medication were independent risk factors for post-operative bleeding. One-year allograft survival was significantly better in the non-bleeding group (91.4% *vs* 75.9%, p=0.023). Multivariable analysis found that post-operative bleeding, recipient age, and biopsy-proven rejection were independent risk factors for graft survival. Recipient age and biopsy-proven rejection were also identified as independent risk factors for patient survival.

**Conclusion:**

This analysis indicated a high risk for post-operative bleeding in renal transplant patients under antiplatelet therapy. The associated negative effect on allograft survival underscored the need to reduce any risk factors for post-operative bleeding.

## INTRODUCTION

The incidence of cardiovascular disease in patients with end-stage renal disease is 10–20-fold that in the general population [1-3]. According to current guidelines, the majority of these patients are treated by inhibition of platelet function using anticoagulant and antiplatelet medications aiming at the reduction of cardiovascular morbidity and mortality [4]. Additionally, anticoagulant and antiplatelet medications are used for hemodialysis access preservation [5].

One side effect of this medication is the increased risk for spontaneous or intervention-related bleeding [6-8]. These adverse events are even more prevalent in case of dual antiplatelet therapy, which is indicated for certain periods after coronary stent implantation or acute coronary syndrome [9-11].

For patients with end-stage renal disease who are scheduled for kidney transplantation, the half-life of these drugs may become a challenge. Time constraints inherent in the field of (deceased donor) transplantation prevent the discontinuation of antiplatelet therapy within a reasonable period before kidney transplantation. Surgery then often proceeds despite recent consumption of medication intake. Therefore, the bleeding risk in these cases is considered to be increased. In some transplant centers, patients on clopidogrel therapy are not considered transplant candidates or are not listed as potential active recipients. So far, the risk/benefit ratio and the impact on the graft outcomes are poorly documented for lack of data.

We conducted this study to investigate the incidence of post-operative bleeding in patients undergoing kidney transplantation with concomitant antiplatelet therapy and analyze the impact on outcome.

## MATERIAL AND METHODS

Study Population

We studied all adult patients who underwent kidney transplantation at the University Hospital Essen, Germany from January 2007 to June 2012. Data were prospectively collected through the Eurotransplant database and the local patient database and retrospectively evaluated for this study. Those recipients receiving clopidogrel, acetylsalicylic acid (ASA) or both were identified and included into the analysis. Patients who underwent multiorgan transplantation and pediatric recipients or patients with oral anticoagulation therapy were excluded. The study protocol was approved by the local Ethics Committee. Due to the retrospective study design informed consent was waived.

The following characteristics were considered for the analysis: donor age, donor body mass index (BMI), kidney donor risk index (KDRI), kidney donor profile index specified for the year 2015 (KDPI), cold ischemia time (CIT), warm ischemia time (WIT), human leukocyte antigen (HLA) A, B, DR mismatches, recipient age, gender, underlying kidney disease, living or deceased donor kidney transplantation, previous kidney transplantation, waiting time from listing to kidney transplantation, cardiovascular disease, time interval since last percutaneous coronary intervention (PCI), number and type of coronary/vascular stents, dual antiplatelet medication, incidence of reoperation for bleeding and vascular thrombotic events, decrease in serum hemoglobin from pre-operative value (ΔHgb) within four postoperative days, red blood cell transfusions, infectious complications, rejection of the allograft, and patient- and death-censored survival. 

All kidneys were transplanted to the right or left iliac fossa and vascular anastomoses were made to the iliac vessels. Ureteroneocystostomy was performed according to Lich-Gregoir. Punch biopsies were collected intraoperatively one hour after reperfusion following careful suture of the kidney parenchyma. Heparin was not administered intraoperatively. Six hours after surgery, prophylactic anticoagulation with unfractionated heparin was started according to the center protocol. Application was closely monitored and adapted to kidney function to prevent accumulation and possible side effects. Antiplatelet therapy with ASA was implemented uninterrupted. A break of clopidogrel therapy for five days from the date of transplantation was performed. Immunosuppression was based on ongoing studies at the time of transplantation and included induction and maintenance immunosuppression. All patients were followed pre- and post-operatively at our outpatient kidney transplant clinic. 

Definition of Delayed Graft Function

Delayed graft function (DGF) was defined as the need for at least one hemodialysis session during the first week post-transplantation.

Definition of Primary Non-function

Primary non-function was defined as permanent loss of allograft function starting immediately after transplantation and handled as graft loss. 

Definition of Post-operative Bleeding 

Bleeding was defined as a serum-hemoglobin (S-HB) decrease of >3.5 g/dL compared to pre-operative baseline S-HB within the first four post-operative days. This definition used along with the Thrombolysis in Myocardial Infarction (TIMI) bleeding criteria, which are one of the most frequently used classifications in cardiovascular patients [12]. A clear definition of bleeding events has not yet been applied to the kidney transplant population. Other studies utilized a much tighter definition of a drop in S-HB of only 2 g/dL with three days post-transplantation [16]. All patients received an adequate infusion therapy intra- and post-operatively. Laboratory examinations excluded any hemodilution or hemolysis. 

Statistical Analysis

Statistical analyses were performed using JMP (version 10.0.0 SAS; SAS Institute Inc., Cary, NC, USA). The distribution of data was tested for normality using one-sample Kolmogorov-Smirnov test. For continuous variables, data are expressed as median and range. Differences in continuous variables were tested using Student’s t test or Mann-Whitney U test. Differences between categorical variables were tested using Fisher’s exact test or χ^2^ test. Univariate and multivariate logistic regression analysis and Cox proportional hazard model were also used. Variables with a p value <0.2 in univariate analysis were included in a stepwise mixed multivariate regression analysis. Risk ratios or odds ratios were obtained from hazard models. The level of missing values for all variables was lower than 5% unless otherwise indicated. Missing values were handled by case exclusion. A p value <0.05 was considered statistically significant. 

## RESULTS

We identified 744 patients who underwent kidney transplantation at our center between 2007 and 2012. Among these, 161 (21.6%) received oral antiplatelet medication and were included in the study. 

Donor Characteristics

The median (range) age of the deceased donors was 56 (2–86) years with a median BMI of 25.7 (12.4–40.4) kg/m^2^. The median KDRI was 1.42 (0.61–3.65) and the median KDPI was 84% (4%–100%). The grafts were transplanted after a median CIT of 12 (1–29.8) hours. The median WIT was 31.5 (11–82) minutes.

Recipient Characteristics

The median (range) age of the recipients was 61 (30–77) years. Among all recipients, 98 (60.9%) subjects were male. Most patients (n=136, 84.5%) were transplanted with a kidney from a deceased donor. Twenty-five (15.5%) patients were transplanted with a living related kidney donor organ. The majority of patients (n=127, 78.9%) underwent their first kidney transplantation, while 27 (16.8%) and 7 (4.3%) patients received their second or third kidney graft, respectively. The median (range) pre-transplant dialysis duration was 36 (0–340) months. 

Due to the inclusion criteria set, this cohort of patients demonstrated a high prevalence of cardiovascular comorbidities. Details are given below. 

Indications for Antiplatelet Therapy

The main indication for treatment with antiplatelet agents was coronary artery disease (n=125, 77.6%). Besides, 25 (15.5%) patients received antiplatelet drugs, in descending order, for peripheral artery disease, atherosclerosis of the carotid artery, atrial fibrillation, and aortic valve sclerosis. In 11 (6.8%) individuals the reason for platelet inhibition was not documented and could not be identified by chart review. 

Seventy-seven (47.8%) patients underwent percutaneous coronary intervention at 17 (range: 2–133) months before transplantation. In 70 (43.5%) cases stent implantation into the coronary arteries was needed. Bare metal stents were utilized in 38 (54%) patients. Drug eluting stents were utilized in 28 (40%) patients and a combination of BMS and DES was used in 4 (6%) patients. History of coronary artery bypass surgery was present in 26 (16.1%) patients. 

Indications for Dual Antiplatelet Therapy

Fifteen patients received dual antiplatelet therapy at the time of kidney transplantation. All but one patient had history of coronary artery disease. The last preceding percutaneous coronary intervention was performed 425 (range: 64–4003) days before kidney transplantation. Two (13%) patients had a drug eluting stent implanted within the last 120 days and therefore a clear indication for dual antiplatelet therapy. Five (33%) patients had a drug eluting stent implanted more than a year before kidney transplantation. In the remaining recipients, bare metal stents were implanted more than 30 days before kidney transplantation. Chart review suggested the following indications for dual antiplatelet therapy: shunt preservation, history of acute coronary syndrome within the last 12 months, dual antiplatelet therapy as alternative to therapeutic oral anticoagulation (warfarin therapy). 

Post-operative Complications

Surgical treatment was required in 37 (23.0%) recipients. Besides bleeding complications (see below), thrombotic complications were identified in 6 (3.7%) patients (thrombosis of the renal vein in two patients, embolism of the renal artery in three, and embolism of the external iliac artery in one patient) and vascular dissection in 2 (1.2%) patients (intima dissection in the external iliac artery in one, and dissection of the donor renal artery in another one patient). 

Biopsy-proven rejections during follow-up were documented in 54 (33.5%) recipients. Other post-operative complications included microbiological proven and symptomatic infections of the urinary tract (n=52, 32.2%) or radiological and laboratory signs of infections of the respiratory tract in 7 (4.3%) cases, lymphoceles in 10 (6.2%) individuals, and major wound healing problems in 54 (33.5%) cases.

Post-operative Bleeding

Post-operative bleeding was observed in 55 (34.2%) of the transplanted patients. Subsequently, transfusions of one unit of RBCs were required in 16 (29%) patients and transfusion of two units of RBCs in 17 (31%) patients. Altogether, 29 (53%) patients needed surgical revision due to bleeding complications. Subcutaneous hematoma was evacuated in 22 (76%) patients. Diffuse retroperitoneal bleeding was found in 7 (24%) cases. 

Risk Factors for Bleeding Complications

Bleeding complications occurred in 31.5% recipients with antiplatelet monotherapy compared with 60% in recipients with dual antiplatelet therapy (p=0.027). Living related kidney transplantation and a lower pre-operative hemoglobin level were identified as protective factors. In contrast, dual antiplatelet medication was associated with a higher risk for post-operative bleeding. Detailed results of the univariate and multivariate analyses are presented in Tables 1 and 2. 

**Table 1:** Univariate analysis of patient characteristics for post-operative bleeding. Values are either median (range) or n (%).

Variable	Bleeding (n=55, 34.2%)	No Bleeding (n=106, 65.8%)	p value
Gender			
Male	30 (54%)	68 (64%)	0.24
Female	25 (45%)	38 (35%)	
Age (yrs)	61 (30–77)	62 (36–76)	0.29
BMI	26.1 (19.1–40.5)	24.2 (17.7–41.8)	0.78
Living related kidney transplantation	4 (7%)	21 (19.8%)	0.03
Number of KT			
First	37 (67%)	90 (84.9%)	0.01
Second	13 (24%)	14 (13.2%)	
Third	5 (9%)	2 (1.9%)	
Dialysis period (month)	40 (0–340)	35.5 (0–326)	0.56
Coronary artery disease	39 (72%)	85 (80.2%)	0.08
Time since last PCI (d)	548 (110–4003)	501 (57–3882)	0.61
Time since first PCI (d)	723 (110–4003)	813 (57–8979)	0.2
Type of coronary stents			
BMS	8 (15%)	30 (28.3%)	0.19
DES	10 (18%)	21 (19.8%)	
History of bypass surgery	8 (15%)	18 (16.9%)	0.69
Preoperative Hgb value (mg/dL)	13.2 (10.5–17.2)	11.9 (9.6–16)	0.0001
ΔHgb (mg/dL)	4.1 (3.5–7.7)	2.4 (2–3.4)	—
Transfusion of RBCs			
Zero units	23 (42%)	105 (99.1%)	0.001
One unit	15 (27%)	1 (0.9%)	
Two units	17 (31%)	0 (0.0%)	
PNF	5 (9%)	1 (0.9%)	0.01
DGF	15 (27%)	21 (19.8%)	0.29
Graft loss	24 (44%)	36 (33.9%)	0.23
Antiplatelet therapy			
Monotherapy	46 (84%)	100 (94.3%)	0.03
Dual therapy	9 (16%)	6 (5.7%)	

BMI: body mass index; KT: kidney transplantation; PCI: percutaneous coronary intervention; BMS: bare metal stent; DES: drug eluting stent; Hgb: hemoglobin; RBC: red blood cell; PNF: primary non-function; DGF: delayed graft function

**Table 2:** Logistic regression analysis for post-operative bleeding

Variable	OR (95% CI)	p value
Living related kidney transplantation	0.16 (0.04–0.58)	0.002
Coronary artery disease	2.29 (1.57–2.77)	0.043
Dual antiplatelet medication	7.04 (1.9–44.5)	0.002

There was no significant difference between patients with and without post-operative bleeding in terms of the donor data (Table 3).

**Table 3:** Univariate analysis of donor characteristics for post-operative bleeding. Values are either median (range) or n (%).

Variable	Bleeding (n=55, 34.2%)	No Bleeding (n=106, 65.8%)	p value
Donor Age (yrs)	57 (2–86)	55 (2–85)	0.955
Donor BMI (kg/m^2^)	25.9 (12.4–40.4)	24.7 (12.4–35.2)	0.733
KDRI	1.42 (0.65–3.38)	1.4 (0.61–3.65)	0.896
KDPI (%)	84 (8–100)	83 (4–100)	0.804
CIT (hrs)	12.4 (0.97–29.8)	12.6 (1.9–28.7)	0.924
WIT (min)	35 (11–67)	34 (15–76)	0.288
HLA A mismatch (0, 1 ,2)	18, 26, 10	26, 26, 26	0.457
HLA B mismatch (0, 1 ,2)	27, 27, 19	42, 15, 47	0.807
HLA DR mismatch (0, 1 ,2)	29, 29, 29	35, 24, 45	0.427

BMI: body mass index; KDRI: kidney donor risk index; KDPI: kidney donor profile index; CIT: cold ischemia time; WIT: warm ischemia time; HLA: human leukocyte antigen

Patient and Graft Outcomes

The median (range) follow-up time was 40 (0–100) months. Delayed graft function occurred in 36 (22.4%) patients. Primary non-function was observed in 6 (3.7%) recipients. 

The patient survival rates after 30 days and 12 months of transplantation were 99.4% and 89.7%, respectively. In multivariate analysis, transfusion of RBCs, recipient age, and biopsy-proven rejections were identified as independent risk factors for patient survival (Table 4). Our definition of post-operative bleeding did not meet the criteria to be entered into the multivariate analysis. However, transfusion of RBCs might serve as a surrogate for blood loss, while other reasons such as low pre-operative hemoglobin, contributed to the transfusion as well. The median (range) ICU and hospital stay for recipients with bleeding complications was 0.8 (0–44.2) and 20.1 (9.4–67.2) days, respectively. Recipients without bleeding complications had a median (range) ICU stay of 0.8 (0–21.4) days (p=0.832) and a significantly (p=0.027) shorter hospital stay of 16.2 (7.6–69.9) days.

**Figure 1:**
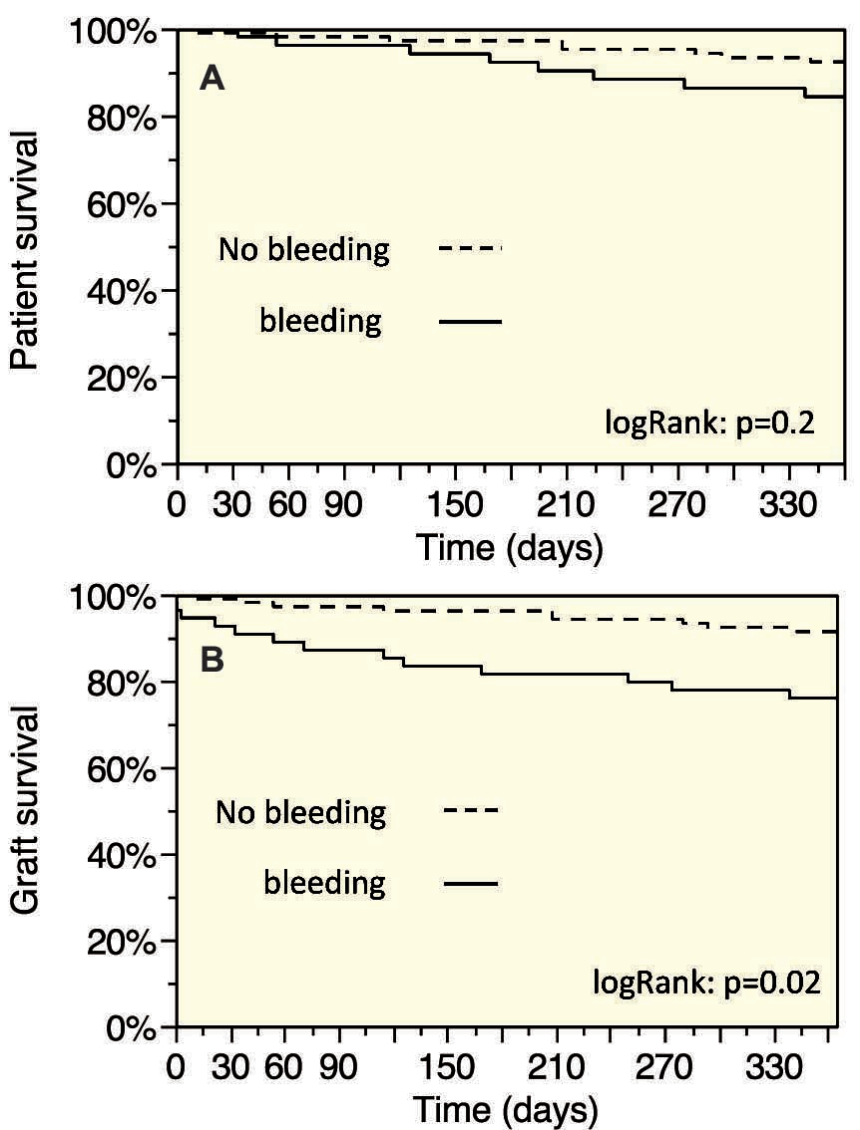
A) Patient and B) survival in dependence of post-operative bleeding

The graft survival rates after 30 days and 12 months of transplantation were 96.9% and 86.1%, respectively. The graft survival rates in recipients without bleeding complications after 30 days and 12 months were 99.1% and 91.4%. In contrast, the rates in recipients with bleeding complications were only 92.7% and 75.9%, respectively (p=0.023). The influence of bleeding complications on graft survival is separately presented in Figure 1. For allograft survival, recipient age, loss of hemoglobin, and biopsy-proven rejection were identified as independent predictors (Table 5). Significance between patients with bleeding and without bleeding episodes remained for the death censored graft survival (p=0.014) in the log-rank test. Further analysis demonstrated that loss of hemoglobin, surgical revision, and biopsy-proven rejection were independent predictors of the death censored graft survival (Table 6). 

**Table 4:** Cox proportional hazard model for the patients survival

Variable	HR* (95% CI)	p value
Age	1.05 (1.01–1.08)	0.005
Transfusion of RBCs	1.49 (0.95–2.2)	0.08
Biopsy proven rejection	1.85 (1.01–3.3)	0.046

* Hazard ratio associated with one unit change in the regressor RBCs: red blood cells

**Table 5:** Cox proportional hazard model for the graft survival

Variable	HR* (95% CI)	p value
Age	1.04 (1.01–1.07)	0.014
Loss of Hemoglobin	1.23 (1.03–1.47)	0.023
Biopsy proven rejection	2.4 (1.41–4.0)	0.001

* Hazard ratio associated with a change of one unit in the regressor

**Table 6:** Cox proportional hazard model for death censored graft survival

Variable	HR* (95% CI)	p value
Loss of hemoglobin	1.37 (1.04–1.8)	0.014
Surgical revision	1.23 (1.02–6.2)	0.023
Biopsy proven rejection	2.4 (1.5–8.9)	0.001

* Hazard ratio associated with a change of one unit in the regressor

Patients who received antiplatelet therapy and developed post-operative bleeding, especially those under dual-antiplatelet therapy, were at a higher risk of suffering primary non-function and a short-term poor outcome.

## DISCUSSION

We evaluated the risk of post-operative bleeding complications in patients undergoing kidney transplantation with concomitant antiplatelet therapy and found risk factors that contributed to this complication. So far, studies investigating the incidence of this complication and its effect on long-term outcome of patients are limited.

In our study, 21.6% of the patients listed for kidney transplantation received antiplatelet therapy. Among them, roughly 10% received dual antiplatelet therapy. Dual platelet inhibition was independently associated with an increased post-operative bleeding. Overall, 18% of patients required surgical treatment to control the bleeding. This resembled a much higher incidence of hemorrhagic complications among patients undergoing kidney transplantation under antiplatelet medication compared to the general kidney transplant population (4%–7%) as reported in the literature [13-15]. 

We found that dual antiplatelet medication, coronary artery disease, and deceased donor kidney transplantation were independent risk factors for post-operative bleeding. This fact become more interesting in the multivariate graft survival analysis where clear sequences of post-operative blood loss was identified as an independent risk factor for graft survival, demonstrating major clinical relevance. Allografts without hemorrhagic complications had a 1-year survival of 91.4% compared to grafts after hemorrhagic complications that had a 1-year survival of 75.9%. This was comparable to data presented by Koch, *et al*, who reported anticoagulation as a risk factor for post-operative bleeding and also an independent risk factor for graft and patient survival [15]. Barba, *et al*, demonstrated an association between the overall surgical complications and donor age, as well as the delayed graft function. However, in their study, donor characteristics were the primary risk factors for vascular and overall and major surgical complications, while recipient characteristics were only wound, urological, and minor complications [13]. Musetti, *et al*, found that antiaggregant therapy is not an independent risk factor for hemorrhagic events after kidney transplantation [14]. Likewise, Hachem, *et al*, reported that pre-operative anticoagulant therapy leads to no significant increase in the risk of bleeding [16].

Renal allograft recipients are becoming increasingly older, owing to long wait times, and therefore have a higher prevalence of diabetes mellitus, hypertension, obesity, and dyslipidemia [17, 18]. These factors are all considered mediators of cardiovascular risk in this population. Therefore, antiplatelet agents are expected to be used commonly in potential kidney transplantation recipients, due to their overall beneficial profiles [19-25], which have been studied extensively.

Although the intake of dual antiplatelet medication was found to be a risk factor for post-operative bleeding, the withdrawal may be associated with stent thrombosis [27]. To reduce the risk of bleeding in the setting of kidney transplantation, the potential recipient could be delisted/inactively listed as long as the dual antiplatelet drug is necessary. Furthermore, being a scheduled procedure, living related kidney transplantation should be acknowledged as a possible alternative to reduce the bleeding risk. If transplantation is carried out in spite of using dual antiplatelet medication, the surgeon should recognize the risk and pay more than usual attention to hemostasis at the end of the procedure.

The use of point-of-care devices, *eg*, multiple electrode platelet aggregometry, may help to identify patients who are at major risk of bleeding by measurement of platelet function [26]. Additionally, the risk for stent thrombosis in patients on dual antiplatelet treatment might be recognized by such diagnostic tools, as shown in a recent study [41].The impedance aggregometry measures the platelet function in whole blood. There are specific assays that can stratify the ASA and/or clopidogrel effect [41, 42]. With the potential of such devices, a thorough risk adjustment seems possible and could help in the process of clinical selection of patients.

Clear indications for dual antiplatelet therapy are usually given in individuals with recent history of acute coronary syndrome as well as in those with implanted bare metal or drug eluting stent [7, 8]. Against this background, the utilization of drug eluting stents and concomitant necessity of long-term dual antiplatelet therapy in patients listed for renal transplantation or potential candidates should be debated. The implantation of bare metal stents with concomitant short-term dual antiplatelet therapy should at least be considered intensively in potential/active renal transplantation recipients. 

While the impact of antiplatelet continuation on intraoperative transfusion requirements is still uncertain, the increased risk in the perioperative period is well documented. Recent studies demonstrated that the combination of clopidogrel and aspirin before various surgical interventions is associated with increased frequency of surgical treatment for bleeding and need for red blood cell (RBC) transfusion [28]. Similar results were shown for the continued use of single antiplatelet therapy carrying additional risk for the patient [29]. However, due to the risk/benefit ratio and the potential prevention of cardiovascular complications the continuation of single antiplatelet medication with ASA is suggested for most abdominal surgeries by the current guidelines [30, 31]. Comparably, minor or no risk elevation has been described in kidney transplant recipients under single or dual antiplatelet therapy [14, 32, 33]. 

Interestingly, our results demonstrated that after bleeding complications, allografts were at higher risk of primary non-function and thus were lost early. This suggested that the bleeding associated hemodynamic alterations and inflammation, as well as anesthesia and surgery associated trauma, would aggravate the recovery from ischemia-reperfusion injury [34-36]. Regenerative capabilities of the allograft might then be increased leading to progressive graft dysfunction and ultimately graft loss. In this context, it might be of interest that neither bleeding events, nor surgical revision, nor transfusion of RBC were associated with an increased risk for allograft rejection in our study (data not shown), whereas previous studies identified allograft rejection as another independent and well-known factor for the allograft survival [37-40]. 

There are some limitations to our study. First of all, the results of retrospective studies are less robust compared to prospective ones. Moreover, it is a unicenter study, which may underlie a local bias and a limited number of patients. A larger sample size would increase the robustness of the statistical results. 

In conclusion, our data indicated a high risk of bleeding in kidney transplant recipients who were treated with antiplatelet medication. Bleeding was associated with a significantly inferior graft outcome. Further studies are needed to determine the indication of using antiplatelet drugs and concomitant diseases. Further studies should address if point-of-care monitoring such as impedance aggregometry may identify patients at risk for bleeding and probably prevent post-operative bleeding episodes.
